# Supplementary material for: Are carbon emissions trading and green financial instruments synergistic? -Comprehensive quantitative research based on content analysis
Source: PLoS One. 2024 Mar 7;19(3):e0298601. doi: 10.1371/journal.pone.0298601 (PMC10919601; doi:10.1371/journal.pone.0298601)
Supplement: S1 Data — (ZIP) [file pone.0298601.s001.zip › Data/地级市Z_F工作报告.zip!/─┌├╔╣┼/╬┌║ú╩╨/└┤╫╘╣1⁄2╓┌║┼í╛┬φ┐╦╩2╛▌═°í┐_2016.docx]

现在，我代表市人民政府向大会报告工作，请连同《国民经济和社会发展十三五规划（草案）》一并审议，并请市政协委员和列席会议的同志们提出意见。

一、十二五时期和2015年工作情况

十二五时期是乌海改革发展进程中极不平凡的五年。面对严峻复杂的经济形势，我们在自治区党委、政府和市委的正确领导下，在市人大、政协的监督支持下，全面落实自治区8337发展思路，坚持实施一个中心、两个转型发展战略，真抓实干，锐意进取，经济社会保持平稳发展。

十二五时期是综合实力持续增强的五年。地区生产总值比十一五末增加218.5亿元，年均增长11.6%。公共财政预算收入是十一五末的2.4倍，年均增长19%。社会消费品零售总额比十一五末增长94%。全社会固定资产投资累计完成1800亿元，是十一五期间的2.7倍，超额完十二五目标任务。

十二五时期是产业结构调整优化的五年。三次产业结构由十一五末的1:71.7:27.3调整为2015年的0.8:60.3:38.9。工业产业延伸升级。煤焦化和氯碱化工两大基地建设成效显著，精细化工快速发展。装备制造、新能源、新材料等非煤产业占工业增加值比重由十一五末的47.7%提高到65.9%。我市成为国家第三批资源型城市转型试点和首批循环经济示范城市。第三产业快速发展。金融业对财政的贡献率由十一五末的3%提高到7.5%，君正、海化在主板挂牌上市。金沙湾被评为国家4A级旅游景区。乌海海关获准设立。现代农业加快发展。葡萄产业不断壮大，葡萄种植面积是十一五末的1.8倍，汉森成为中国驰名商标。

十二五时期是城市基础设施建设力度不断加大的五年。累计投入基础设施建设资金570亿元，是十一五期间的2.2倍。建成区面积由37.5平方公里扩大到62.3平方公里。黄河海勃湾水利枢纽工程建成蓄水，乌海湖118平方公里水面逐步形成。我市被评为国家智慧城市试点、信息惠民国家试点城市、全国第七个住宅产业化综合试点城市。立体交通网络日益完善，公路总里程达931公里，乌海机场旅客吞吐量达180万人次。大力实施保障性安居工程，开工建设各类安置房9.3万套，4.6万户居民居住条件得到有效改善。当代中国书法艺术馆建成开馆，教育、文化、卫生等公共基础设施达到自治区先进水平。

十二五时期是生态环境持续改善的五年。累计投入绿化资金40多亿元，实施重点林业和园林绿化工程371项。建成区绿化覆盖率达到42.2%，人均公园绿地面积达到19.7平方米。我市成为全国绿化模范城市和国家园林城市。海勃湾北部生态涵养区保护管理成效明显。投入大气污染治理资金84亿元，集中开展主要污染物减排等重点环境治理工程，全面完成;十二五减排任务。实施淘汰落后产能项目40个，单位GDP能耗比十一五末下降16%，万元工业增加值用水量比十一五末下降36%。

十二五时期是人民生活水平不断提高的五年。民生和社会事业年均支出占公共财政支出的70%以上。城乡居民人均可支配收入分别为十一五末的1.72倍和1.7倍。城镇新增就业4.5万人，托底安置就业困难人员8500人，零就业家庭实现动态清零。我市成为首批国家级创业型试点城市。社会保障提标扩面，低保、医保、养老保险、住房保障等方面实现城乡同等待遇，低保标准等主要民生指标居自治区前列。我市成为全国首批全民参保登记计划试点城市。实现了小学到高中十二年免费教育。公立医院改革成为国家和自治区试点。

各位代表，回顾十二五，特别是刚刚过去的一年，我们主动适应经济发展新常态，全力以赴稳增长、调结构、促改革、惠民生，经济社会保持平稳健康发展。全年地区生产总值完成609.82亿元，增长7.5%。公共财政预算收入完成80.48亿元，增长7.1%。固定资产投资完成398.64亿元，增长13.2%。社会消费品零售总额完成139.06亿元，增长7.4%。城镇和农村牧区常住居民可支配收入分别达33968元、14402元，增长7.9%、7.3%。

一年来，我们顶住经济下行的巨大压力，把抓项目、扩投资作为稳增长的第一举措，定向施策、精准发力，实现了经济稳中有进、稳中向好。

抓政策落实。及时出台了拓宽融资、援企稳岗和扶持中小微企业等60余项具体措施。协调争取电力优惠政策，28户企业进入电力多边交易范围，为企业减少电费支出1.3亿元。协调金融机构采取续贷、延期等措施，帮助困难企业解决资金近百亿元。减免、缓收涉企税费4亿元。落实援企稳岗政策，认定困难企业18户，涉及参保人数2.4万人，补贴资金6673万元。降低失业保险缴费率，失业保险费率由3%降到2%，全年为企业减负1437万元。

抓项目建设。认真落实自治区重大项目三年三级实施计划，实行一个项目、一套班子、一套方案、一抓到底，拟建项目抓审批、在建项目抓进度、建成项目抓投产，20个自治区级重大项目全部开复工，115个市级重点建设项目综合开复工率达到74%。

抓投融资创新。推进政府与社会资本合作，储备PPP项目87个，9个项目列入国家PPP项目库，乌海湖大桥纳入国家开发银行PPP项目试点。建立市投资集团、漠海绿洲农业发展公司等融资平台，组建产业引导基金、城乡一体化发展基金等6支基金，总规模263亿元。设立8000万元转贷基金，为企业提供资金支持20多亿元。加大招商引资力度，全年到位资金241.8亿元。争取上级各类资金30亿元，为稳定经济增长提供了有力保障。

一年来，我们解放思想、凝聚共识，把改革创新作为引领发展的第一动力，打破利益格局，破除体制障碍，实现了发展环境的进一步优化。

大力推进简政放权、放管结合。实行一个部门管市场。整合工商、质监、食品药品监管职能，成立市场监督管理委员会，构建横向到边、纵向到底统一监管的市场管理新格局。实行一个机构管城市。整合各部门城市管理职能，成立城市管理委员会，城市管理工作由多头管理向集中管理转变，形成统一指挥、部门联动的大城管格局。实行一个窗口管审批。审批事项全部进驻政务服务大厅，所有行政审批实现一站式服务，95%的行政审批事项现场办结。完成三证合一、一照一码登记制度改革，新增市场主体8636户，增长56.3%。非公经济持续健康发展，对固定资产投资贡献率达61%。实行一个平台管服务。建立覆盖市、区、街道、社区四级的政务服务信息平台，民生业务实现网上申报、跨区转办、并联审批。12345和110实现两线联动服务，便民服务水平进一步提高。实行一个清单管权力。建立权力清单制度，公布市本级行政权力4251项。启动责任清单编制工作。

不断强化科技创新支撑作用。全社会科技研发经费投入占地区生产总值比重达到1.2，连续两年位居全区首位。恒业成和乌海化工被认定为国家级高新技术企业，阳光田宇和宝化万辰获得自治区重大科技专项支持，海勃湾工业园区、网讯信息科技有限公司被列为自治区首批众创空间试点。

一年来，我们直面困难、砥砺前行，把转型升级作为经济发展的第一要务，实现了产业转型新进展。

工业转型迈出新步伐。焦炭气化战略取得突破，面对煤焦行业市场低迷的发展困局，鼓励焦化企业寻求新的发展路径，源通、华信通过实施LNG项目逐步走出了转型发展新路子。依托传统产业优势，大力发展精细化工，佳瑞米草铵膦、天津亚东萘系列项目试产，源宏医药中间体一期进展顺利。装备制造业取得新进展，中钰镁合金轮毂项目签约落地。新兴产业规模化水平进一步提升，天宇化工高档填料一期基本完工，国华光伏发电一期并网运行，青岛昌盛日电太阳能项目开工建设。乌达工业园区被评为国家新型工业化产业示范基地。

现代服务业比重进一步提升。第三产业增加值完成237.3亿元，增长9.7%，占GDP比重由32.8%提高到38.9%。物流业改革创新试点有序推进，物流业占第三产业增加值比重达20%。成立乌海湖旅游公司和黄河明珠文化旅游产业投资公司，兰亭广场码头、游客服务中心等项目进展顺利，乌海湖水上娱乐、蒙根花温泉水世界等项目投入运营。乌海湖休闲度假旅游区被列入2015年全国优选旅游项目名录。全年接待游客190万人次，增长15.7%；实现旅游收入32.3亿元，增长25%。内蒙古股权交易中心乌海办事处挂牌成立，百联汇、国泰君安等金融企业入驻我市，银行业贷款余额高于存款余额110亿元，保持资金净流入。万达广场、浙商国际等商贸综合项目进展顺利，大宗商品交易中心投入运营，40家电商企业入驻创客空间，电子商务等新型业态发展活跃。对外贸易总额达到4000万美元，增长122%。

以葡萄为主的特色农业加快发展。启动葡萄及葡萄酒产业十三五规划编制工作。乌海葡萄地理标志证明商标成功注册，葡萄品牌影响力不断扩大。以葡萄种植、深加工为主的一二三产业相融互促发展，葡萄种植面积达到3.2万亩，葡萄酒销售收入2.2亿元。休闲农业企业达到12家，其中5家成为国家和自治区级休闲农业示范点。蔬菜种植面积稳定在1.6万亩。万亩滩盐碱地改造工程基本完工。海南区土地确权登记颁证试点进展顺利，流转农区土地2万余亩。龙头企业和农区居民利益联结机制进一步完善，带动7000户农区居民实现增收。

一年来，我们夙兴夜寐、主动作为，把十个全覆盖作为统筹城乡发展的第一工程，加强城市规划建设管理，实现了城乡环境的全面改善。

十个全覆盖工程稳步推进。累计投入10.9亿元。完成街巷硬化167.7公里、危房改造6092户。建成海北新城饮水安全工程，解决了1.2万农区居民安全饮水问题。村文化活动室、便民超市等6项提标提质工程进展顺利，行政村文化综合服务中心实现全覆盖。开展村容村貌整治，建立综合管理、环境卫生、医疗等城乡一体化服务长效机制，农区公共服务水平明显提高。

城市规划建设水平不断提升。编制完成国民经济与社会发展十三五规划，启动工业转型发展、园区基础设施、旅游业发展等专项规划编制工作。加大城市基础设施建设力度，硬化街路16条、14万平方米。甘德尔河三期等城市水系建设稳步推进，新建人工湖6个，形成5.5公里城中水系景观。海勃湾食品加工园区投入运营。乌达、海南生活垃圾处理厂投入使用，我市被列入国家第五批餐厨废弃物资源化利用和无害化处理试点。完成交通建设投资15.3亿元，千里山与蒙西园区道路改建工程竣工，下穿包兰线立交桥建成通车。我市成为国家新能源汽车试点城市和自治区首家全国绿色交通城市试点。

全国文明城市创建取得阶段性成果。我市入选第四届全国文明城市提名城市。大力实施百日攻坚行动，集中开展交通秩序、文明餐桌、诚信建设等一系列专项整治活动，集中整治集贸市场24个，城区面貌明显改善。充分发挥媒体作用，深入宣传中国梦、社会主义核心价值观和文明礼仪。实施文明网络、文明家庭等创建主题工程，文明新风日益深入人心。

生态环境不断改善。投入社会资金45亿元，切实解决环境污染问题。组织实施主要污染物减排项目97个，预计削减二氧化硫2.2万吨、氮氧化物2.7万吨。电力企业全部实现达标排放，焦化企业污染防治走在全国前列。淘汰炼铁、水泥等落后产能175.4万吨。启动实施5项矿区环境综合治理示范工程。十三五规划战略环评列入自治区十三五规划。联合编制完成《乌海及周边区域污染防治规划》，与石嘴山市签署了《区域环境污染联防联控合作协议》。组织实施了海惠公路、乌海湖右岸等绿化工程，建成区新增绿地面积20.6公顷。全面落实最严格水资源管理制度，三条红线指标全部达到自治区控制标准。完成摩尔沟河道治理、小流域综合治理等工程15项，全国水生态文明城市试点建设通过国家中期评估。我市成为全国第二批生态文明先行示范区。

一年来，我们坚持以人为本、执政为民，把保障和改善民生作为政府工作的第一目的，不断加大民生投入力度，实现了人民生活的持续改善。

民生保障不断强化。推进大众创业、万众创新，创客空间等5个创业园和创业孵化基地入驻创业实体587户，带动就业1500人。城镇登记失业率控制在自治区下达目标以内。不断完善社会保障体系，城镇职工基本养老、失业、工伤、生育保险参保率持续提高。最低生活保障标准提高到585元，企业退休人员基本养老金提高到月人均1996.2元，增长10.5%。扎实推进保障性安居工程建设，完成投资9.7亿元，新开工建设19910套，建成7048套。积极发展养老事业，建成市老年人养护服务中心。大力实施公交便民惠民政策，补贴资金2670万元。深入推进精准扶贫，制定出台了覆盖就业、养老等10个领域的帮扶政策措施，实现对口帮扶全覆盖，全年脱贫805人。新建供热站13座。海勃湾南部4万吨净水厂建设进展顺利。创新物业管理方式，启动10个智慧小区试点。

社会事业全面进步。实施第二期学前教育三年行动计划，新建改建幼儿园5所。推动义务教育均衡发展，高中教育办学质量不断提高，高考本科上线率63.2%，高于自治区平均水平15个百分点。职业技术学院试办本科专业2个。在自治区率先全面推开城市公立医院综合改革，公立医院全部实施药品零差率销售，减轻群众用药负担2300多万元。分级诊疗试点初显成效，逐步实现双向转诊，急慢分治。加强与北京协和医院等医疗机构的技术交流合作，医疗水平持续提升。市妇幼保健院通过国际JCI三年复审。设立文化产业发展专项基金，实施文化产业重点项目27个。成功举办第七届黄河明珠•中国乌海书法艺术节暨第四届国际书法产业博览会等大型活动，承办自治区文化旅游融合发展系列活动，广场文化艺术节等文化惠民活动蓬勃开展。举办环乌海湖全国竞走大奖赛等7项全国性赛事，10所学校成为国家级特色足球学校。

一年来，我们创新治理、齐抓共管，把法治作为政府工作的第一原则，把稳定作为政府工作的基本保障，社会保持和谐稳定。

全面推进依法治市。自觉接受市人大及其常委会和市政协的监督，按时办结人大代表建议149件、政协委员提案148件。广泛征求和吸纳各民主党派、社会各界对政府工作的意见建议。深入推进依法行政，建立政府法律顾问制度，组建了法律顾问专家库。建立重大决策合法性审查、重大行政决策听证制度，推进重大行政决策风险评估机制。完善依法行政评议考核机制，健全纠错问责程序。加大对公务支出的审计力度，实现公共资金、国有资产、国有资源和领导干部履行经济责任情况审计全覆盖。深化政务公开，市区两级预算决算和三公经费支出全部向社会公开，全年压缩市本级三公经费18.5%。扎实开展三严三实专题教育活动。

社会治理成效显著。社区民生服务平台不断完善，率先在全区实现社区民生数据实时采集。不断深化平安乌海建设，深入开展新一轮十小平安创建活动。完善立体化治安防控体系建设，实现了重点场所、新建小区技防全覆盖。建立公共安全周会商研判机制，公共安全形势总体良好。畅通网上信访渠道，全市信访总量持续下降。落实安全生产政府监管责任和企业主体责任，强化危险化学品、煤矿及非煤矿山、食品药品等重点领域安全专项检查，安全生产形势总体稳定。支持工会、共青团、妇联等人民团体开展工作，关心残疾人工作，监察、审计工作得到加强，国防建设、民族宗教、档案史志、外事侨务、人防、气象、防震减灾、新闻出版、广播影视、无线电管理和红十字会工作取得新成绩。

这些成绩的取得，是自治区党委、政府和市委正确领导、科学决策的结果，是市人大、市政协加强监督、鼎力支持的结果，是全市广大干部群众守望相助、团结奋斗的结果。在此，我代表市人民政府，向全市广大干部群众和所有关心支持乌海建设发展的各界人士，致以崇高的敬意和衷心的感谢！

二、十三五发展思路和2016年工作安排

十三五是我市转变经济发展方式的关键期、全面深化改革的攻坚期、全面建成小康社会的决胜期。十三五时期我市经济社会发展的总体要求是：全面贯彻党的十八大、十八届三中、四中、五中全会精神，深入落实习近平总书记系列重要讲话特别是考察内蒙古重要讲话精神，以四个全面战略布局为统领，坚持创新、协调、绿色、开放、共享发展理念，贯彻自治区8337发展思路和党委九届十四次全委会议精神，认真落实市委六届十一次全委会议精神，深入实施一个中心、两个转型发展战略，全力推动生态立城、形态塑城、业态兴城、文态铸城、动态创城五态一体发展，突出民生优先、绿色优先、旅游优先，更加注重人才保障、更加注重创新驱动，全面建成小康社会，建设富裕、美丽、文明、和谐、幸福乌海，打造祖国北疆亮丽风景线上的璀璨明珠。

十三五时期我市主要预期目标是：地区生产总值预期年均增长8%左右，固定资产投资年均增长11%，公共财政预算收入年均增长7%，社会消费品零售总额年均增长10%，城镇常住居民人均可支配收入年均增长8%以上，农村牧区常住居民人均可支配收入年均增长9%以上。城镇新增就业人数4.5万人，城镇登记失业率控制在自治区下达目标以内，居民消费价格涨幅控制在3%左右，单位生产总值能耗指标完成自治区控制任务。到2020年，实现地区生产总值、城乡居民收入比2010年翻一番，全面建成小康社会。

围绕以上目标，我们将重点打造四大基地、五大中心。

区域清洁能源输出基地。完善能源基础设施，推进阿拉善、乌海华中电力外送通道建设，争取进入上海庙山东电力外送通道。发挥地区资源优势，积极发展低热值煤发电、光伏发电以及抽水蓄能、瓦斯发电、尾气发电，打造电力洼地。到2020年，新能源装机比例达到30%以上。调整优化能源输出结构，大力发展LNG等清洁能源，积极争取国家支持进入西气东输管网，将资源优势转化为经济优势。

全国重要的煤焦化工、氯碱化工和精细化工产业基地。加快焦化企业兼并重组，扩大产业集群效应和行业整体竞争力。实施焦炭气化战略，实现资源分质分级利用，不断延伸煤焦化工、氯碱化工产业链条，发展以农药医药中间体、多元醇、PVC糊树脂等产品为主的精细化工产业。

区域循环经济示范基地。围绕主导产业，加强上下游企业间的配套链接，形成行业间、园区间和区域内的循环系统。大力发展危废处理、污水处理等环保产业，推动电石渣、粉煤灰等固体废弃物资源化利用，加快发展新型节能环保墙体材料等建材产业，建设资源节约型、环境友好型社会。

以葡萄产业为主导的现代农业基地。坚持按照全产业链模式发展现代农业，大力发展葡萄种植和葡萄酒加工，构建一区、一带、三圈、百庄发展格局，打造中国•乌海沙漠原生态葡萄酒庄之都。依托国家一带一路发展战略，探索建设葡萄酒交易平台，加强葡萄酒文化对外交流。

区域文化旅游中心。全力做好山、水、城三篇文章，发挥地区特色资源优势，打造中国西部旅游集散中心和国际特色旅游目的地。围绕山，重点打造以蒙元文化、草原文化、赏石文化为特色的甘德尔山生态文明景区。围绕水，以沿黄生态文明产业带开发建设为重点，着力打造集观光旅游、体育竞技、休闲度假、会议会展于一体的乌海湖黄金岸线。围绕城，充分发挥六张名片品牌优势，着力打造生产空间集约高效、生活空间宜居适度、生态空间山清水秀的沙地绿洲、水上新城。

区域健康产业中心。依托市人民医院滨河分院和雅泰高科技产业园，大力发展以医疗服务、药品器械、耗材产销应用为主体的医疗产业。深入挖掘蒙医蒙药、温泉沙疗等资源，大力发展健康理疗产业。建设为老服务平台，大力发展满足多样化养老服务需求的养老产业。

区域大数据应用中心。以政务云中心为核心，建设以一云、两网、四库、+应用为主要架构的智慧乌海。实施互联网+行动计划，推动工业、旅游、金融、城市管理等10个领域与互联网深度融合发展。积极培育大数据应用市场，推动区域社保、医疗、教育、养老、就业、公共安全、社区服务等领域信息资源互通共享。

区域金融服务中心。大力引进各类股份制金融机构，支持乌海银行等地方金融机构做大做强，鼓励通过企业上市、发行债券、股份转让、股权交易等方式直接融资，建立多层次资本市场体系。扶持证券、期货及新型金融业态发展，构建立足乌海、辐射周边的金融服务体系。

区域生产性物流中心。完善交通运输网络，积极促进呼银高铁建设，实施乌海机场扩建。力争到2020年，全市公路里程达到1200公里。推动互联网+物流，发展面向乌海及周边地区的第三方专业仓储配送、出口代理、技术咨询、员工培训、检验检测等生产性服务业。依托乌海海关，发展跨境电子商务，提高对外贸易水平。

各位代表，十三五的发展蓝图已经绘就，关键在于落实。2016年，我们将按照市委六届十一次全委会议的安排部署，围绕农区十个全覆盖和脱贫攻坚工程、产业结构调整、生态环境保护、棚户区改造、创建文明城市五项重点工作，以更务实的作风、更有效的举措，加快推进经济转型和城市转型，建设自治区西部区域中心城市，向着全面建成小康社会的宏伟目标奋勇前进。

2016年，我们的主要预期目标是：地区生产总值增长8%，规模以上工业增加值增长8%，公共财政预算收入增长7%，全社会固定资产投资增长12%，社会消费品零售总额增长10%，城镇常住居民人均可支配收入增长8%，农村牧区常住居民人均可支配收入增长9%，节能减排完成自治区约束性控制指标，居民消费价格涨幅控制在3%以内，城镇登记失业率控制在自治区下达目标以内。

围绕以上目标，全市干部群众必须解放思想、深化认识、克服困难、闯关过坎、锐意改革、大胆创新。面对新常态，必须按照习总书记提出的十个更加注重要求，按照五大发展理念，落实五大政策，瞄准结构性改革特别是供给侧改革，依靠市场，转换动能，厚植产业发展优势，着力在去产能、去库存、去杠杆、降成本、补短板上下功夫、求实效。重点做好以下六方面工作。

（一）加快推进产业转型，提高经济发展质量和效益

紧紧抓住自治区打造乌海及周边地区资源型产业转型发展示范区的有利契机，下大力气推动工业转型升级，厚植工业发展新优势，培育转型发展新动能。大力实施焦炭气化战略。引导煤焦企业通过焦炭气化形成循环经济体系，鼓励企业延伸发展甲醇、烯烃等下游产业链，实现产业要素优化重构。重点扶持源通、华信焦炉煤气制LNG项目投产达效，发挥引领带动作用。积极促成华油天然气与现有焦化企业合作新建焦炭制气项目，启动恢复生产。围绕清洁能源基地发展，启动输气管网等配套基础设施建设。引导焦化企业进一步提高产品质量，增加焦炭品种，充分发挥现有产能。抓住中蒙深化合作的战略机遇，依托乌海海关，扩大对外开放，拓宽焦炭销售市场。延伸优化氯碱化工产业。以科技创新为动力，着力打通煤焦、氯碱化工相融互促、循环发展通道。依托神雾集团聚乙烯项目，发挥其生产工艺技术优势，推动氯碱化工产业转型升级、多元发展。积极承接京津冀产业转移，推进天津渤海精细化工、渤天化工项目落地开工，充分发挥其资金、品牌和市场优势，推动精细化工向集群化、终端化发展。培育壮大新兴产业。依托陕汽新能源汽车带动配套产业集群发展，加快中钰镁合金轮毂、陕汽零部件配套产业园等项目建设，推动轻便直升机等装备制造项目尽早落地开工，促进产业集聚发展。大力发展光伏产业，围绕鼎晶光伏、青岛昌盛日电太阳能等项目，规划建设光伏电站。加快推进新能源汽车试点建设，做好抽水蓄能电站建设前期工作。联合科研院所和高新技术企业，成立环保产业研发中心，鼓励发展高效节能、资源循环利用的节能环保产业，着力培育新的经济增长点。

（二）加快推进城市转型，打造区域服务中心

建设区域生产性物流中心。依托鸿达物资和PVC交易中心等电商平台，搭建支付结算网络。重点抓好乌海北货场、宜嘉药品仓储中心等物流项目建设，积极引进国内知名电商企业在我市建立仓储配送中心。建设区域金融服务中心。组建市土地资源收储投资公司，探索土地开发融资新模式。完善金融服务体系，引进股份制银行等金融机构，筹建金融控股集团等投融资征信服务平台，扩大金融服务范围。扶持市投资集团、乌海银行、汉森等本地企业上市。金融对财政贡献率保持在8%以上，市内外贷款余额与GDP比率不低于120%。加强国有资产资本化运作，引导更多的社会资本进入基础设施和公共服务领域。做好政府存量债务置换、民间借贷监管和风险案件处置，有效防范金融风险。建设区域文化旅游中心。以乌海湖黄金岸线为核心，以甘德尔山生态文明景区和乌海湖休闲旅游度假区为重点，加快推进乌海湖旅游码头、乌海院子、高山草甸等项目建设，提升金沙湾、蒙根花等旅游项目建设营运水平，打造中国西部旅游集散中心和国际特色旅游目的地。我们推进旅游优先战略，不仅要依靠旅游的硬实力，更要发挥文化的软实力，让书法文化、蒙元文化、草原文化、赏石文化成为乌海人的精神家园。依托当代中国书法艺术馆、书法广场举办各类会展节庆赛事等活动，举办黄河明珠•中国乌海书法艺术节，提高中国书法城建设水平。以环乌海湖国际马拉松邀请赛、全国自行车和桥牌等大型赛事为带动，举办乌海国际户外节，打造房车营地、沙漠越野、低空飞行等休闲旅游活动品牌。积极推进户外装备制造产业园前期工作，建设中国户外运动城。规划建设以甘德尔山、乌海湖为核心，以山体、水体、桥体为重点的亮化工程，打造山、河、湖通体亮化旅游景观，使夜景成为乌海文化旅游特色品牌。建设区域健康产业中心。启动健康产业园规划建设，依托市人民医院滨河分院和雅泰高科技产业园，大力发展医疗服务、理疗旅游、休闲保健等健康产业。启动市老年养护服务中心公建民营改革试点，建设为老服务中心。鼓励社会力量兴办养老机构，扶持康复护理、居家养老等服务业发展，构建医养护一体化服务体系，真正实现老有所养。建设区域大数据应用中心。大力实施互联网+10大行动计划，依托政务云中心，加快建设数据交换平台。完善智慧乌海民生服务平台，在社区实现一张表格采集、一个平台录入、一个出口共享，实现80%以上社区业务网上办理，社会保障一卡通覆盖全部户籍人口。实施地名标识工程，加快智慧交通、智慧小区等信息工程建设。

提升城市规划建设管理水平。突出规划的引领作用，树立以人为本、尊重自然、传承历史、绿色低碳的规划理念，修改完善城市总体规划，推进经济社会发展规划、城乡规划、土地利用规划、城市电网规划等多规合一改革，形成全市一本规划、一张蓝图。全面加强城市建设。统筹城市地下和地上、旧城和新城、城区和园区基础设施建设，着力提升城市综合承载能力。大力推进城市综合管廊规划建设，建设地下综合管廊11.8公里。加快推进旧城改造，改造供水、供气、供热等管网494公里、老旧小区60个，新改建公厕129座，继续抓好既有建筑节能改造。实施海勃湾南220千伏输变电工程等城市配电网项目，启动君正和懿峰电厂热电联供工程。整合优化部门职能职责，推进水务一体化改革。完善交通网络，新改建东环路、光明路等城区道路21条，力争打通中心城区所有断头路。开工建设巴音赛互通立交桥，完成乌海湖大桥西引线、石嘴山黄河大桥东引线及国道244巴音陶亥镇南段工程，力争乌海湖大桥年内建成，乌海至宁东沿黄高速公路等项目早日开工。建成海勃湾南部4万吨净水厂，开工建设海勃湾北部10万吨和乌达、海南净水厂，确保居民饮用水安全。完善工业园区水、电、路、蒸汽、污水及固废处理设施，提升综合配套服务能力。加强职业教育基础设施建设，推动职业技术学院升格应用型本科院校。建成职业病防治院、精神卫生中心和精神智障人员福利院，完成图书馆、体育中心足球场改造，加快职业技能公共实训中心一期、蒙中医院二期建设，新改扩建幼儿园4所，启动博物馆、生命纪念园等项目规划建设。切实强化城市管理。以全国文明城市创建为抓手，按照严管重罚、注重引导的原则，不断巩固和深化交通秩序、市容市貌等专项整治的成果。坚持协调协同，积极引导企业、社会组织和广大市民投身城市管理，使政府有形之手、市场无形之手、市民勤劳之手同向发力，共同集聚城市发展正能量，实现城市共治共管、共建共享。

（三）以十个全覆盖为统领，全面提升城乡一体化水平

带着对农区居民的诚挚感情，带着干事创业的激情，全力以赴推进十个全覆盖工程，把农区打造成为乌海城市转型的新亮点。

推进十个全覆盖工程。以十个全覆盖工程为带动，促进城市基础设施向农区延伸、公共服务向农区拓展、城市文明向农区辐射。加大投入力度，同步全面推开行政村、自然村、涉农社区、城乡结合部十个全覆盖工程，在7月底前全面完成农区十个全覆盖工程建设任务。编制完善村庄布局规划，大力实施移民搬迁工程，高标准、高水平、高质量打造黄河沿线示范新村。实行建设质量终身负责制，建立农区基础设施和工程管理长效机制。按照一村一品、一村一业原则，结合沿黄生态文明产业带规划建设，大力发展农家乐、渔家乐、观光采摘等休闲农业，实现人居环境、自然环境和经济效益、生态效益的有机统一。

推动农业与二、三产业融合发展。把葡萄全产业链发展模式作为一二三产融合发展的示范，充分发挥产业引导基金的带动作用，鼓励农业龙头企业加大葡萄种植力度，规范葡萄质量标准，推进云飞、田野等葡萄标准园建设。鼓励发展红酒温泉疗养产业，集中建设一批特色酒庄酒堡，努力把汉森、阳光田宇、吉奥尼等酒庄建设成为国家4A级旅游景区，全力打造中国·乌海沙漠原生态葡萄酒庄之都。充分发挥乌海葡萄地理标志证明商标品牌价值，推动葡萄产业向中高端市场迈进。举办丝绸之路中国·乌海沙漠葡萄酒文化节，提高我市葡萄酒品牌知名度。加快农区土地流转，完善龙头企业与农区居民利益联结机制，促进农业产业规模化、农区生产工厂化，实现富农增收。抓好菜篮子工程建设，加快闲置温室整合利用，大力发展设施农业，力争蔬菜自给率达到27%。合理布局农贸市场和蔬菜直销店，支持农业龙头企业建立集种植、配送、销售于一体的蔬菜供销体系，有效稳定供应价格。依托农资、农产品配送网络，发展农村电商。

（四）突出绿色优先，打造生态安全屏障

全面开展环境综合整治。进一步加大矿区环境综合治理力度，完成439处煤矸石自燃和24家露天煤矿排土场整治，硬化道路120公里以上。所有焦化企业实现达标排放，新建燃煤发电机组严格执行超低排放限值，对现有燃煤发电机组进行超低排放改造。全部淘汰10蒸吨及以下燃煤锅炉，建筑工地全部达到绿色施工要求，工业园区及城区周边企业物料堆场实现全封闭。完成十三五规划战略环评编制，全力推动大气污染联防联控制度的落实，切实改善区域大气环境质量。深入实施水污染防治行动计划，工业园区污水处理厂全部实现达标排放。建设骆驼山矿区和三区工业园区空气质量自动监测站，建成空气质量自动监测网络。

着力推进生态文明建设。将沿黄资源优势转化为产业优势，完善黄河沿线基础设施建设，打通沿黄生态旅游公路，加快发展葡萄深加工、光伏农业、养殖业等生态高效特色产业，着力打造沿黄生态文明产业带。加快国家森林城市创建工作，大力推进京津风沙源治理、天然林保护等国家重点林业工程，突出抓好甘德尔生态综合治理二期、骆驼山矿区植被恢复、乌海湖右岸、葡萄公园二期、龙游湾湿地堤坝两侧等造林绿化工程。深入挖掘林业经济效益增长点，吸引社会资本投入生态建设。积极推进全国水生态文明城市试点建设，加强集中式饮用水水源地保护，启动地下水超采区治理。研究制定海勃湾北部生态涵养区保护和管理条例、乌海市湿地保护条例，力争通过人大立法形式予以颁布。加快构建生态文明制度体系，加强生态文明宣传教育，营造爱护生态环境的良好社会风尚。

（五）突出民生优先，让人民群众共享发展成果

实施创业就业工程。进一步完善落实创业就业政策措施，鼓励支持创业带动就业。设立高校毕业生就业创业引导资金，实施大学生就业促进计划和创业引领计划。加强创业园和创业孵化基地建设，鼓励发展互联网+新业态，激发大众创业、万众创新活力。大力提升技能培训实效，持续加强定向、定岗培训，培育具有区域特色的培训品牌和精品专业，打造符合市场需要的就业技能实训基地。做好就业援助，帮助就业困难群体实现就业。年内新增城镇就业7500人以上，失业人员再就业3000人，完成培训7900人次以上。

实施社会保障工程。进一步完善社会保险、城乡低保、社会救助衔接联动的城乡社会保障体系，织密民生保障网。深入实施全民参保登记计划，建立社保扩面征缴与劳动用工备案联动机制。探索实行市级统筹、分级负担的医保基金管理体制。加快推进机关事业单位养老保险制度改革，重点抓好农区居民养老保险参保工作。全面加强低保、医疗救助、特困人员供养等社会救助工作。

实施百姓安居工程。按照政府主导、市场运作的原则，积极引入央企等社会资本参与，加快推动城市棚户区改造，确保早日实现百姓安居梦。计划投资129亿元，实施海勃湾东山二期、下海勃湾、乌达五虎山和海南拉僧庙等17个棚户区改造项目，着力解决3万户以上的棚户区居民住房问题。认真落实去库存要求，结合城乡一体化和农区人口转移，推进棚改货币化安置，推动建立租购并举的住房制度，加快消化房地产库存。

实施教育质量工程。优化教育布局，提高办学水平，着力促进教育公平发展和质量提升。完成第二期学前教育三年行动计划目标任务，力争适龄幼儿入合格园率达到80%。推进校长教师定期交流，促进义务教育均衡发展，确保海勃湾区义务教育均衡发展通过自治区和国家评估验收。深化普通高中特色课程建设，提升高中教育办学质量。规范发展民办教育，加快发展职业教育、民族教育、特殊教育。

实施医疗卫生提升工程。扎实推进医改工作，巩固完善基层医改成果，加快公立医院综合改革步伐。优化整合医疗资源，建设以市人民医院为核心、覆盖所有基层医疗机构的医联体；推广完善分级诊疗模式，制定促进分级诊疗的差异化医保支付政策，着力解决人民群众看病难、看病贵、基本医疗卫生资源配置不均衡等问题。加强区域卫生信息平台建设，推行诊疗一卡通，实现医疗机构就诊信息共享。完善药品集中采购制度，探索跨区域联合采购。

实施文体惠民工程。完善公共文化服务体系，承办好第26届全国图书交易博览会系列活动，继续开展广场文化艺术节、全民阅读等群众文化活动。出台文化产业发展规划，建设文化产业示范基地。开展书法万里行活动，提升乌海特色文化影响力。全面推进足球改革工作，推广沙滩足球、笼式五人制足球项目。广泛开展全民健身运动。

实施物业服务提质工程。加强物业服务管理，推广建设100个智慧小区。不断拓展物业服务覆盖面，切实改善小区物业特别是老旧小区环境和物业服务质量。强化部门责任落实，建立物业企业统一登记备案制度，加大对物业企业的监管力度。探索机关单位物业企业与小区物业企业合作模式，全面提升物业服务水平。编制社区综合管理服务责任清单，明确政府职能部门、街道办事处、社区和水、电、气等专业服务单位在住宅小区综合管理中的职责，推动政府管理和公共服务进小区。

实施集贸市场建设工程。全面加强集贸市场建设，新建集贸市场5个，完成提标改造16个。健全完善市场管理常态化机制，加大市场环境综合整治力度，为群众营造整洁、舒心、便利的市场环境。按照划行归市到位、环境管理到位、设施建设到位的要求，促使集贸市场做到有序经营、诚信经营、文明经营。

实施食品药品安全工程。建设食品药品安全监管平台和追溯体系，创建国家食品安全城市。构建食品快速检验检测体系，大型集贸市场全部具备食品快速检测能力。强化食品药品监管信息化工程和应急体系建设，提升突发事件和重大事故应急反应水平。

实施脱贫攻坚工程。完善精准扶贫机制，实施产业扶贫一批、教育扶贫一批、生态扶贫一批等五个一批工程，完成年度扶贫任务811人，实现贫困人口两不愁、三保障。

严格落实安全生产责任制，加快建设安全生产隐患动态监管与风险防御信息化系统，防范和遏制重特大安全事故发生。全力做好信访维稳工作，健全矛盾纠纷排查化解机制，综合运用司法调解、人民调解、仲裁等手段，畅通群众诉求表达渠道。加强法治乌海建设，深入推进七五普法。严厉打击各类违法犯罪活动，构建立体化社会治安防控体系，建设平安乌海。抓好自治区成立70周年和建市40周年迎庆工作。充分发挥工会、共青团、妇联等人民团体的桥梁纽带作用，关心支持妇女儿童、残疾人、红十字会、慈善救助等事业，加强民族宗教、国防建设、双拥优抚、人防、气象、地震、消防、档案史志、新闻出版、广播影视、无线电管理等工作。

（六）强化保障措施，确保各项工作任务落到实处

过去的一年，虽然我们付出了比以往更多的努力，但与预期仍有一定差距，这里既有经济下行的因素，也有我们自身努力不到位的问题。部分干部对新常态认识还不到位、不适应，对政策的研究还不到位，把握上级政策导向、抓政策落实的能力还有待提高。我们要坚持党的领导，坚持人民主体地位，把群众利益作为一切工作的根本出发点和落脚点，以改革创新为动力，以人才资源为支撑，把加强自身建设作为最有力的保障，解放思想，真抓实干，确保各项工作落到实处。

更加注重改革创新。推进简政放权、放管结合，重点抓好行政审批、投资审批、收费清理、商事制度等领域改革，进一步优化服务环境。规范完善权力清单，加快责任清单编制工作。全面开展不动产统一登记工作，实现多证合一。鼓励社会中介组织规范发展，充分发挥商会、协会作用，有序承接政府职能转移。完成公务用车制度改革。加快公交体制改革，提升便民服务水平。依托煤焦化工和氯碱化工研究院等平台，引导企业加大投入，着力打造国家级研究院和检验检测中心。

更加注重人才资源。充分发挥企业、科研院所、高等院校等各领域专业人才作用，聘请知名专家学者和行业领军人物，加快建设乌海智库。加强人才队伍建设，积极引进各领域专业人才，为转型发展提供人才保障和智力支持。出台加强和创新人才工作的实施意见，设立人才火炬计划等专项资金，营造人才成长发展的良好环境，确保各类人才进得来、留得住、用得好。

更加注重自身建设。坚持科学施政。广大干部要深刻认识三期叠加背景下的新形势、新常态，认真学习和研究国家、自治区的新政策、新判断，对不适应、不适合甚至违背新的发展理念的认识要立即调整，对不适应、不适合甚至违背新的发展理念的行为要坚决纠正，对不适应、不适合甚至违背新的发展理念的做法要彻底摒弃。坚持务实勤政。始终保持奋发有为的精神状态，主动作为，准确研判和把握宏观经济形势，全力抓好各项政策落实，加强对企业的指导与服务，切实将政策红利转化为现实生产力。践行三严三实要求，大力弘扬立说立行、即说即办的工作作风，敢闯敢试、敢于担当、敢于负责，把更多的时间和精力用到工作落实上来。把抓落实的成效作为衡量干部能力的标准，完善考核问责机制，大力整治不作为、慢作为、乱作为。坚持依法行政。把依法行政作为提升政府公信力的重要途径，将所有工作纳入法治化轨道。严格执行《预算法》，强化预算支出约束。健全法律顾问制度，落实重大行政决策法定程序，依法接受人大法律监督、政协民主监督和社会舆论监督。坚持廉洁从政。全面落实党风廉政建设主体责任，严守廉洁从政各项规定。健全规范和制约权力运行的制度体系，完善土地出让、产权交易、工程招标、政府采购等公共资源交易体系。加强对重点领域不正之风的专项治理，加大对违法违纪案件的查处力度，真正做到干部清正、政府清廉、政治清明。

各位代表，目标鼓舞人心，蓝图催人奋进。让我们在市委的正确领导下，紧紧依靠全市人民，同舟共济，破浪前行，为全面建成小康社会而努力奋斗！
